# Protein suppresses both bitterness and oleocanthal-elicited pungency of extra virgin olive oil

**DOI:** 10.1038/s41598-021-91046-0

**Published:** 2021-06-04

**Authors:** Catherine Peyrot des Gachons, Abigail J. O’Keefe, Louise Slade, Gary K. Beauchamp

**Affiliations:** 1 Monell Chemical Senses Center, Philadelphia, PA USA; 2 Food Polymer Science Consultancy, Morris Plains, NJ USA

**Keywords:** Neuroscience, Physiology, Psychology

## Abstract

The Mediterranean diet, considered one of the healthiest in the world, is characterized in part by the major source of its fat, which is extra virgin olive oil (EVOO). Among the health benefits of consuming EVOOs is the presence of phenolic compounds, which have been shown to lower the incidence of coronary heart disease and are suspected of providing many other health benefits. These phenolic compounds also contribute to the flavor of EVOO, adding both specific pungency in the throat and bitter notes that are valued by connoisseurs but reported to be unpleasant by naïve consumers. Here, we demonstrate that some food-derived proteins, specifically from egg yolks and whey, when added to pungent and bitter EVOOs, reduce or even eliminate both the throat pungency and bitterness. The sensory loss is proportional to the food protein additions. Thus, when used in various foods recipes (e.g. mayonnaise), pungent and bitter EVOOs may lose their pungent and bitter characteristics thereby rendering them more palatable to many consumers. This sensory reduction might also indicate interaction between the proteins and the phenolic compounds, which, if confirmed, would raise the question of whether the bioactivities of EVOO phenolics remain unchanged when consumed with and without protein-containing foods.

## Introduction

Extra virgin olive oil (EVOO) is central to the Mediterranean diet, which is considered one of the healthiest in the world^1^. The consumption of EVOO has been associated with many positive nutritional properties, including a well-established lower incidence of coronary heart disease^2–4^. Major beneficial constituents of EVOO are the phenolic compounds, which other vegetable oils lack, particularly the phenolic alcohols and secoiridoid derivatives^5^. The health promoting properties of EVOO phenolics are linked to their anti-inflammatory, anti-oxidant and anti-microbial activities. These phenolic compounds are also associated with immune system regulation, modulation of metabolism and reduction of several oxidative and inflammatory related diseases^6^.

**Figure 1:**
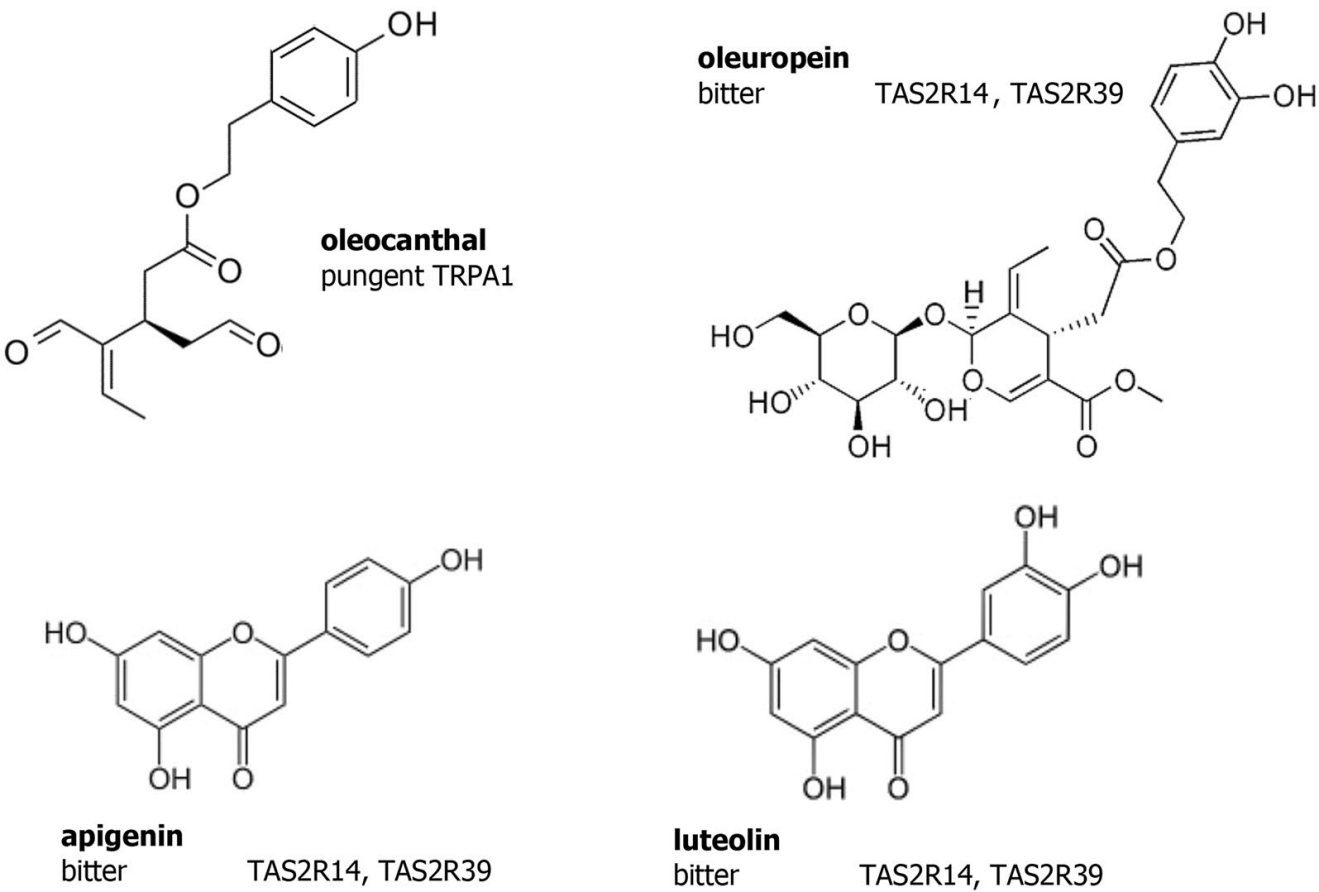
Chemical structures of endogenous compounds present in Extra-Virgin Olive Oil with their perceptual descriptors and the taste and sensory receptors by which the perceptions are mediated: oleocanthal (pungency in the throat, but not bitter), oleuropein (bitter, but not pungent), apigenin (bitter, but not pungent), and luteolin (bitter, but not pungent).

The phenolic compounds in certain EVOOs are also responsible for two of their characteristic sensory attributes: bitterness and pungency^7^. Indeed, when consumed neat, many EVOOs taste bitter and evoke an irritation primarily sensed in the throat, which may trigger throat clearing and cough. Although pungency and bitterness are sometimes considered variations of one sensory attribute, they are actually elicited by two separate sensory pathways and often by different compounds. In the case of EVOO, the concomitant perception of both sensations only reflects the simultaneous presence of different compounds, some of which are perceived as bitter such as oleuropein or apigenin and, one primarily, the secoiridoid derivative oleocanthal (OC), an αβ-unsaturated dialdehyde phenolic compound, that is perceived as pungent in the throat^8,9^. Bitter tasting compounds bind to a specific and large set of oral taste receptors, the TAS2Rs, comprised of 25 putatively functional members in humans^10^. Small phenolic compounds found in plant products such as EVOO are known to taste bitter via the activation of several of these TAS2Rs^11–13^ (Fig. 1). Pungency, in contrast to bitterness, is a chemesthetic percept, which derives from stimulation of some non-neuronal cells and free nerve endings in the skin and, most importantly for food perception, in the mouth, throat and nose. Chemesthesis conveys the sensations of pain from very hot water, the burn from chili peppers or the tingling of Szechuan leaves. Most of the known irritant receptors belong to the TRP channel family^14^. In the case of EVOO, the characteristic throat pungency is largely mediated via the activation of TRPA1 by OC^15^ (Fig. 1).

The throat pungency of many highly valued EVOO varieties is well known and appreciated among many of those who evaluate and savor EVOOs in their unadulterated liquid form. In the official European EVOO scoring system for sensory evaluation, this characteristic pungency or irritation is one of only three positive sensory attributes^16^. However, many casual consumers of these oils seem unaware of their irritating properties. Although, there are many possible explanations for this unawareness, one is that EVOOs consumed in foods may not provoke the same level of throat irritation as they do when consumed alone, without food. Supporting this supposition, Dinella et al.^17^ showed a clear reduction of both perceived pungency and bitterness of olive oils mixed in uncooked canned tomatoes and beans. The main focus of their study was on how addition of EVOO to vegetable foods might impact the sensory profile and liking of the vegetables, and not the reverse. Therefore, no further experiments were conducted to clarify the mechanism underlying the reduction of pungency and bitterness observed. Since only 10% of olive oil (w/w) was added, simple dilution could have been the predominant factor in the perceived intensity decrease. However, Dinella et al. also listed several other possibilities including chemical–physical interactions between sensory active molecules in olive oils and food matrix compounds. This latter hypothesis is consistent with ensuing results published by the same group^18^. They reported that when a phenol extract from waste water produced during production of olive oil was added to several foods, subsequent recovery of phenols was reduced as were sensory properties including bitterness and buccal pungency. Also consistent with this interpretation, Pripp et al.^19^ reported reduced bitterness perception of an olive oil phenolic extract in the presence of sodium caseinate and weak binding between the extracted phenolic compounds and some proteins.

These intriguing suggestions in the literature could explain the apparent absence of realization among many naïve consumers that EVOOs are often quite pungent and bitter. Moreover, they were consistent with results of pilot sensory studies we conducted using pungent and bitter EVOOs to make mayonnaises. In pilot studies, we observed large reductions in throat pungency and bitterness of these simple model mayonnaises made with high OC-containing EVOOs. This pilot work, together with the few literature reports, led us to design a series of experiments to more formally demonstrate the profound suppressing effects of protein on both throat pungency and bitterness of EVOOs. In addition to the implications these results have for sensory and culinary aspects of EVOO use, they also raise important questions about the bioavailability and bioactivity of presumably healthful EVOO phenolics when consumed with many foods. OC in particular has been receiving a lot of attention in recent years; it is believed to be a potent anti-inflammatory agent with high preventive and/or therapeutic potential for several human illnesses such as certain cancers, neurodegenerative diseases and rheumatic diseases^2^.

## Results

### Model mayonnaises made with pungent and bitter EVOOs are not perceived as pungent or bitter (Experiment 1)

**Figure 2:**
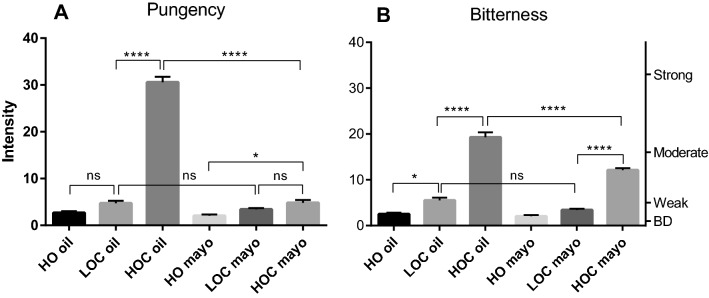
Comparison of perceived throat pungency (**A**) and bitterness (**B**) from 3 oils: HO (High Oleic acid Safflower oil), LOC (EVOO low in OC) and HOC (EVOO high in OC), and from 3 model mayonnaises made with the corresponding oils and 8 g egg yolk/100 g oil. The right axis displays the corresponding portion of the LMS scale used by participants to provide their ratings (BD = barely detectable). 10 subjects (3 females and 7 males; average age 28) rated each sample 4 times in random order. Data are represented as mean + /- SEM. Statistics: ns = not significant; *p < 0.05; **p < 0.005; ***p < 0.001; ****p < 0.0001.

To investigate the potential effects of food constituents on EVOO perception, we first asked participants to evaluate the throat pungency and bitterness intensity of pure oils, presented in liquid form and three model mayonnaises made with the corresponding oils. The first oil, a non-EVOO oil (HO), was a high oleic acid safflower oil, chosen to be the best match to EVOO regarding its lipid composition. The oil is highly refined to remove all but trace amounts of any components but triglycerides. It displayed little or no pungency or bitterness (Fig. 2). The second oil was an EVOO containing a low amount of OC (39 mg/kg of oil), and was weakly pungent and bitter. In these studies, it is referred to as LOC (Low OC). The third oil was an EVOO containing a high amount of OC (349 mg/kg of oil), was very pungent and moderately bitter, and referred to as HOC (High OC). To prepare a model mayonnaise from each of the oils, 100 g of oil, 9 g of pH 7 bottled water and 8 g of egg yolk were combined. None of the additional ingredients often used in traditional mayonnaise recipes such as salt or lemon were used in our samples in order to limit gustatory stimulations other than those coming from the oils themselves and to avoid potential effects of mixture suppression by those ingredients. The six samples were all evaluated by the subjects within a testing session.

Focusing on the results of the 3 oils evaluation first, Fig. 2A shows that participants judged the HOC oil strongly pungent (30.5 intensity rating), whereas HO and LOC oils were perceived as being only weakly pungent (2.6 and 4.7 respectively) and they were not significantly different from each other in intensity (*p* = 0.172). In contrast, the HOC oil was much more pungent than either of these oils (*p* < 0.0001). These results are consistent with previous work^8^ indicating a strong association between the amount of OC in EVOO and the oil level of throat pungency. The HOC oil was also judged clearly more bitter (19.3) than HO and LOC oils (2.5 and 5.5 respectively; *p* = 0.0041 between HO and LOC and *p* < 0.0001 for both oils compared to HOC) (Fig. 2B) which is most likely due to higher content of bitter tasting phenolic compounds such as those mentioned in Fig. 1 in HOC oil (523 mg/kg total phenols as tyrosol equivalents) compared to LOC oil (201 mg/kg total phenols as tyrosol equivalents).

Strikingly, when HOC was tested as a model mayonnaise (HOC mayo; Fig. 2A), its pungency almost entirely vanished (4.8 rating, *p* < 0.0001 compared to HOC oil). This mayonnaise was made of 90% oil and thus dilution cannot be responsible for the large loss in pungency intensity (-84%) relative to the pure HOC oil. The observed pungency suppression most likely reflects the fact that OC is no longer able to activate the TRPA1 receptors through which the sensation is mediated^15^. Similarly (Fig. 2B), perceived bitterness of the HOC mayonnaise decreased markedly, although to a lesser extent (-37%) (rating 12.1, *p* < 0.0001 compared to HOC oil).

### Egg yolk free model mixtures made with pungent and bitter EVOO maintains pungency and bitterness (Experiment 2)

The results of Experiment 1 could be due to partition of OC and other bitter phenolics to some of the egg yolk components (the most functional being, protein (16%), lecithin (7%), cholesterol (1%) and water (50%))^20^. Alternatively, it could be due to the sequestration of OC and the bitter tasting compounds in the egg mayonnaise emulsion.

To study the observed phenomenon further, in Experiment 2, we prepared a water/oil emulsion displaying similar consistency to the model mayonnaises used in the first experiment, but free of egg yolk and thus of its components. For that, we replaced the egg yolk with a surfactant blend consisting of 0.4 g of Tween 80 and 0.6 g of Span 80 (see “Materials and Methods” section for details). Participants evaluated the throat pungency and bitterness intensity of three model mixtures without egg yolk, each made with a different oil (HO, LOC and HOC).

**Figure 3:**
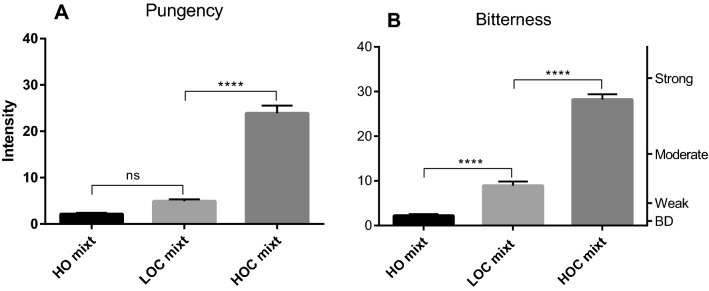
Perceived throat pungency (**A**) and bitterness (**B**) from 3 egg yolk free model mixtures made with 3 different oils and a surfactant blend: HO (High Oleic acid Safflower oil) mixture, LOC (EVOO low in OC) mixture and HOC (EVOO high in OC) mixture. The right axis displays the corresponding portion of the LMS scale used by participants to provide their ratings (BD = barely detectable). 10 subjects (4 females and 6 males; average age 28) rated each sample 4 times in random order. Data are represented as mean + /- SEM. Statistics: ns = not significant; *p < 0.05; **p < 0.005; ***p < 0.001; ****p < 0.0001.

In contrast with the perceptual loss observed with the HOC mayonnaise in Experiment 1, the pungency (Fig. 3A) and the bitterness (Fig. 3B) of the model mixtures made with HOC oil was maintained. Indeed, in Experiment 1, LOC and HOC mayonnaises showed comparably low levels of pungency and were not distinguishable (*p* = 0.588), whereas in this experiment, pungency intensities of LOC mixture and HOC mixture were clearly different (*p* < 0.0001); they were much more aligned to the perceived intensities reported for the corresponding oils in liquid form (Experiment 1), with HOC oil triggering higher pungency than LOC oil. Similarly, the bitterness perception was not suppressed in the HOC mixture. The conclusion of Experiment 2 is that the pungency and bitterness reductions observed in the first experiment in the HOC mayonnaise were not replicated in an emulsion made without egg yolk. These results are consistent with the hypothesis that the suppression of EVOO pungency and bitterness observed in the mayonnaise samples is attributable to the presence of egg yolk and not due to a physical entrapment of the sensory compounds in the emulsion droplets.

### Reduction in EVOO pungency in model mayonnaise is positively correlated to the amount of egg yolk used (Experiment 3)

Experiment 3 was designed to evaluate quantitative differences in the suppression of pungency and bitterness in the presence of egg yolk. To accomplish this, we prepared four HOC mayonnaises with increasing amounts of egg yolk (1, 2, 4 and 8 g per 100 g of oil). For reference, many mayonnaise recipes recommend the use of 1 large egg yolk for 150 g of oil; this would correspond to approximately 12 g of egg yolk per 100 g of oil.

**Figure 4:**
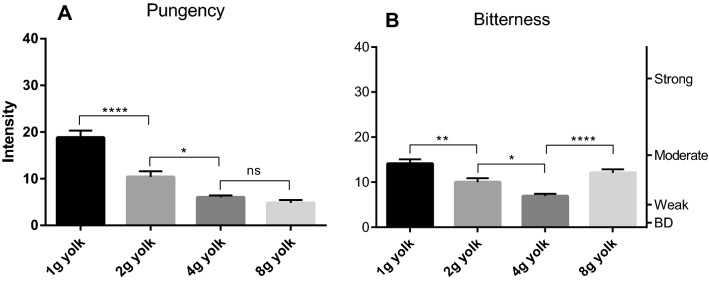
Perceived throat pungency (**A**) and bitterness (**B**) from model mayonnaises made with HOC (EVOO high in OC) and increasing amount of egg yolk (g/100 g oil). The right axis displays the corresponding portion of the LMS scale used by participants to provide their ratings (BD = barely detectable). 10 subjects (4 females and 6 males; average age 33) rated each sample 4 times in random order. Data are represented as mean + /- SEM. Statistics: ns = not significant; *p < 0.05; **p < 0.005; ***p < 0.001; ****p < 0.0001.

The results of this experiment were straightforward for pungency perception. The more egg yolk incorporated into the model mayonnaise the less pungent it was perceived (Fig. 4A). Indeed, at 4 to 8 g egg yolk per oil sample, the pungency almost completely vanished. The relationship between bitterness intensity reduction and increasing amount of egg yolk was less apparent than with the throat pungency but the egg yolk appears to decrease bitterness perception as well (Fig. 4B). The bitterness intensity evaluation obtained here at 8 g seems more complex than the simple monotonic suppression of throat pungency. Egg yolk appears to decrease bitterness perception at lower levels, but the convolution of three different emulsifiers of egg yolk (protein, lecithin—promotes oil in water emulsion, and cholesterol—promotes water in oil emulsion) may affect the behavior of egg yolk at the highest level tested (Fig. 4B). To remove this possible confounding factor, protein alone was evaluated in increasing amounts with a constant level of surfactants in the samples.

### Food proteins in model mixture cause major EVOO pungency and bitterness loss in a dose response manner (Experiment 4)

Proteins are reported to bind to phenolic compounds. Since protein is a major component of egg yolk (16%), we hypothesized that they play the major role in EVOO pungency and bitterness suppression in model mayonnaise. In Experiment 4, testing this supposition, participants evaluated the same model mixture as used in Experiment 2 (emulsion of EVOO high in OC with fixed concentration of surfactants), but here the test samples were prepared with increasing amounts of pure protein isolate. The goal was to determine whether the addition of pure protein isolate would reduce pungency and/or bitterness perception of the HOC mixture.

A whey protein isolate, characterized by a high content of proteins (> 95% protein on solids basis, of which 65% was β-lactoglobulin and 25% was α-lactalbumin) with a high dissolution factor in water was incorporated into our model mixture in increasing amount (0, 0.25, 0.5, 0.75 g and 1.0 g protein per 100 g of HOC oil). Pungency and bitterness of the four samples were evaluated by participants as previously described.

**Figure 5:**
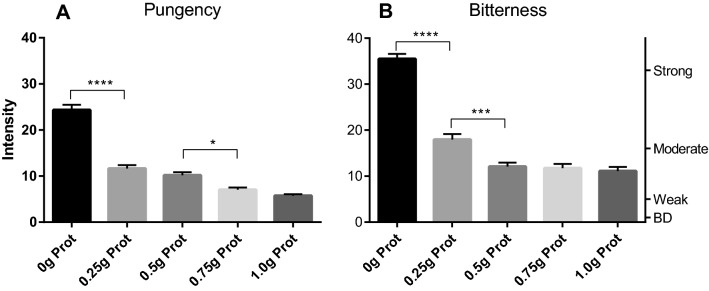
Perceived throat pungency (**A**) and bitterness (**B**) from mixtures made with HOC (EVOO high in OC), surfactants and increasing amount of whey proteins (0, 0.25, 0.5, 0.75 and 1 g/100 g oil). The right axis displays the corresponding portion of the LMS scale used by participants to provide their ratings (BD = barely detectable). 10 subjects (4 females and 6 males; average age 36) rated each sample 4 times in random order. Data are represented as mean + /- SEM. Statistics: ns = not significant; *p < 0.05; **p < 0.005; ***p < 0.001; ****p < 0.0001.

The results of this experiment were similar to those of Experiment 3 in which increasing amount of egg yolk were used. Increasing amount of proteins in the test samples elicited a reduction in perceived pungency and bitterness in a dose dependent manner (Fig. 5). A small addition of whey protein (0.25 g) into the mixture already produced a strong effect on the perceptual qualities of EVOO, with a loss of 52% pungency and a loss of 49% bitterness compare to the control (0 g protein). Additional proteins, with all other variables held constant, further suppressed both perceived pungency and bitterness. These results support the hypothesis that the presence of food proteins in the sample trigger sensory reduction due to interaction between the proteins and both OC (throat pungency inducing) and bitter tasting EVOO compounds.

## Discussion

Based on the studies described, we suggest that protein interactions with phenolic compounds best explain the dramatic loss of pungency and bitterness when small amounts of protein are added to pungent and bitter extra virgin olive oils. Mixture suppression is highly unlikely to explain the profound reduction in pungency and bitterness since the added protein amounts are small relative to the amount of oil and have mild sensory properties on their own. Yet, this conclusion that OC and other phenolics bind to the proteins thereby reducing their chemesthetic and taste properties is based on inference rather than direct proof of binding. Nevertheless, the data presented are consistent with the hypothesis that OC and other phenolics bind to the proteins studied. One caveat to this conclusion might be that in the experiments described added protein was always accompanied by the addition of surfactant materials. Could surfactants be responsible for the sensory inhibition rather than the proteins? We believe this is unlikely since Experiment 4 demonstrated protein dose–response effects on sensory inhibition while concentrations of the added surfactants were held constant. As protein was added the inhibitory effects increased (Fig. 5) in concert with the added protein while the surfactant concentrations did not change. This implicates the protein as the variable most responsible for the pungency and bitterness inhibitory effects. We cannot at this time rule out a cooperative role for the surfactants in facilitating protein binding but we conclude, pending further ongoing experiments, that protein binding accounts for the majority of the sensory inhibitory effects observed.

The data presented here are consistent with the fact that phenolic compounds are known to interact with proteins through hydrogen bonding, hydrophobic and hydrophilic interactions^21^. We have thus tentatively concluded that observed sensory suppression in presence of food proteins is caused by the binding of the free sensory compounds to proteins. We propose that the formation of such complexes make the pungency-inducing and bitterness-inducing EVOO compounds no longer available to activate their respective sensory receptors (TRPA1 and TAS2Rs). In the case of OC, the major source of throat pungency in EVOOs, beside interactions via its phenolic moiety, the molecule can form adduct with proteins via electrophilic addition between the dialdehyde moiety of the molecule and the protein side chains such as the ε-amino groups as present in lysine, thiol groups present in cysteine, or the indole group of tryptophane^21^. We have previously shown that any modification of the unsaturated dialdehyde moiety of OC impairs its TRPA1 activation, which mediates its pungency^15^. Therefore, such modification of the aldehyde groups would also result in the sharp reduction of perceived pungency observed in our experiments. Another possibility is that an OC-protein complex or a bitter phenol–protein complex cannot access the binding sites on TRPA1 or TAS2Rs because of the excessive size of the protein complexes. The binding sites of TAS2Rs are buried in the transmembrane portion of the bitter taste receptors^22^, and the activation of TRPA1 by OC does not depend on canonical binding to intracellular cysteine residues^15^, rather OC activation of TRPA1 may involve lysine residues^23^ or noncanonical binding in the transmembrane portion of TRPA1^24^.

Only few studies on olive oil phenolic compound and protein interactions have been published. Most of them used either EVOO phenolic extracts, which has the disadvantage of possible compound loss and structural transformations during extraction, or pure single phenols known to be present in olive oils. In one study, Genovese et al.^25^ assessed the effect of olive oil phenolics on the release of aroma compounds from an oil/water emulsion stabilized by whey protein. They observed an increased release of some volatile compounds in presence of EVOO phenolic extracts. They hypothesized that less protein binding sites were available for hydrophobic interactions with aroma compounds due to the fact that whey proteins concomitantly interacted with EVOO phenolics. Quintero-Florez et al.^26^ examined interactions between EVOO phenolic compounds and gastric mucin (at pH 3.5 and 37 °C). Via turbidity measurements, they observed formation of insoluble complexes between EVOO phenolic compounds and mucin after 1 min of reaction, the extent of which depended on the phenol concentration and chemical structure. Pripp et al.^19^ studied the effect of sodium caseinate on bitterness perception of olive oil phenolic extract. They found that adding 1% (w/v) sodium caseinate, in presence of Tween 60, to an oil-free or a 65% oil in water system caused a reduction in bitterness. No mention of perceived pungency was made, most likely because their EVOO phenolic extract did not contain oleocanthal. In another publication, they later studied binding of olive oil phenolic compounds to several food proteins including sodium caseinate, bovine serum albumin, β-lactoglobulin and gelatin. In their experiments, not all the phenolic compounds tested bound to proteins and β-lactoglobulin did not bind to the phenolic extracted, which is inconsistent with the results in the experiments reported here. They suggested there was a relatively weak affinity (compared to tannic acid) between proteins and EVOO phenolics^27^ and they predicted that the effective reduction of bitterness perception when in mixture would be limited. De Toffoli et al.^18^ investigated the sensory impact of three plant-based foods (bean puree, potato puree and tomato juice) when mixed with a phenolic extract from olive oil waste water, which apparently did not contain OC. They reported results generally consistent with our findings that food materials reduced bitterness of phenolics found in olive products. However, their methodology precluded investigation of the distinctive throat irritation induced by OC, which was a focus of our research. Our study demonstrates for the first time that along with bitter tasting phenolic compounds, OC in the presence of some proteins leads to a profound loss of the specific EVOO throat pungency in certain foods. The food preparation steps will also have an impact, since other food components can also interact and proteins can be denaturated by heat. The rate and nature of interactions will ultimately trigger different perceptual effects.

Interactions between EVOO phenolic compounds and proteins could potentially affect their bioavailability and physiological activities. EVOO phenolic compounds can interact with proteins at several locations and stages: (1) with food during food processing as discussed, but also, (2) in the course of digestion in the oral cavity, pharynx and the gastrointestinal tract with food proteins, enzymes and microbiome, (3) after absorption, with plasma proteins, and finally, with target proteins in different tissues of the body. Indeed, multiple studies have demonstrated the involvement of OC and other EVOO phenolic compounds in the modulation of several pathogenic pathways via interaction with specific protein targets located in different tissues^2^. The locations of the protein-phenol interactions, the OC target location (tissues), the mode and location of the OC activation site on the target will all dictate the biological activity of the compound. The nature of the OC-protein interaction (reversible or irreversible, chemical groups involved) is another key element which can impact the activity of both partners, phenolic and protein^21^. For example, it has been demonstrated in vitro that OC abrogates fibrillization of tau protein by locking tau into the naturally unfolded state. It has therefore been suggested that OC has the potential of becoming a novel therapeutic agent for neurodegenerative tauopathies such as Alzheimer’s disease^28^. Structure–function studies demonstrated that the two aldehyde groups of OC are required for the inhibitory activity^29^. This echoes the mode of activation of the ion channel TRPA1 by OC^15^. In both cases, if the aldehyde groups are not intact, at the moment of interaction, OC cannot activate the protein target. However, it is also well known that agonist association with proteins, before ingestion or in in vitro assays, does not predict pharmacological efficacy^30^. In vitro assays lack the metabolism, the transport and the movement among compartments of the dynamic in vivo systems^31^. Association with proteins is considered to decrease first pass metabolism for functions where the agonist passes through the bloodstream and liver, for example. So again, the protein interacting effects of oleocanthal and other EVOO phenolics on pharmacology functions will depend on the target of the health benefit.

The perception of pungency and bitterness sensations are greatly valued by olive oil connoisseurs as a sign of high-quality olive oil, but when general consumers undergo preference tests between various olive oils, they most frequently select the oils with low bitterness and pungency^32,33^. Inducing people to consume EVOOs high in polyphenols for health purpose might therefore be a difficult task especially in emergent markets less familiar with those sensations. However, drinking EVOO on its own is rare among consumers. Olive oil is a versatile ingredient with a wide range of cuisine uses such as dressing for cold and hot dishes, as a recipe and marinating ingredient, and as a cooking oil for roasting or frying^17^. As the data reported illustrate, the sensory profile of EVOOs will vary depending on its use in the kitchen and the components of the food matrix to which it is added. Most importantly, consumers need to understand that bitterness and pungency levels might greatly decrease once in their dish^34^.

## Materials and methods

### Participants

A total of 20 people (8 females and 12 males) between 20 and 55 (mean 30) years of age were recruited from the Philadelphia area and were paid to participate in one or more of the studies described here. Each sensory experiment was conducted with 10 participants (average age and gender proportion for each experiment is provided in the corresponding figure legend). All subjects were healthy with no known taste or smell deficiency. Pregnant women and people with egg allergies or under medication for diseases that are known to interfere with taste were excluded from the study. Subjects were asked to refrain from eating and drinking one hour before all experimental test sessions. The study protocol was approved by the Institutional Review Board at the University of Pennsylvania (IRB #7). All research was performed in accordance with relevant guidelines/regulations, and participants gave written informed consent prior to participation.

### Stimuli

Extra-virgin olive oils (EVOOs) were purchased at MillPress Imports, Bethlehem, PA, USA. The EVOO with low pungency was made of Picual olives from California (201 mg/kg total phenols as tyrosol, analyzed by HPLC-UVD and 39 mg/kg OC analyzed by TLC). The EVOO with high pungency was made of Coratina olives from Italy (523 mg/kg total phenols as tyrosol, analyzed by HPLC-UVD and 349 mg/kg OC analyzed by TLC). High oleic acid (HO) safflower oil (Oléico brand), whey protein isolate (biPro brand from Agropur), organic eggs and pH 7 bottled water (Volvic brand) were commercially purchased. Tween 80 (CAS 9005-5-6) and Span 80 (CAS 1338-43-8) were purchased at Millipore-Sigma.

### Sample preparation

The procedure to prepare the test samples was standardized as follows: a total of 100 g of oil was mixed to 9 g of water in presence of egg yolk (8 g in Experiment 1; 1 to 8 g in Experiment 3). Specifically, the addition of the initial 33 g of oil was done dropwise at a constant rate using a separatory funnel attached to a ring stand. This was immediately followed by constant slow stream for remaining 67 g of oil while continually mixing with a commercial kitchen mixer (Hamilton Beach model bowl rest). In all the other experiments, egg yolk was replaced by a single concentration of surfactants in all test stimuli: 0.4 g Tween 80 premixed with water and 0.6 g Span 80 premixed with oil. In Experiment 4, where the samples also contain whey proteins (0.25 to 1.0 g), the protein isolate was added to the water first followed by Tween 80.

### Sensory test protocol

All the samples were made two days before testing and stored in a refrigerator at approximately 5 °C. Samples were brought to room temperature before testing. Eight to 12 samples presented in medicine cups (5 ml for oil samples and 2 g for mayonnaise or mixture samples) were tested per session, with a 4 min break between each sample. All the samples of a given study were tested at least twice within a session. Subjects were asked to rinse their mouth 3 times with water and drink a small amount before each test sample. They were then asked to fully consume the sample in two stages (swallow half and then, 1–2 s later, swallow the second half) and then immediately evaluate the level of throat irritation and bitterness on a labeled magnitude scale (LMS)^35^. The LMS requires subjects to rate the perceived intensity along a vertical axis lined with adjectives: barely detectable (BD) = 1, weak = 5, moderate = 16, strong = 33, very strong = 51, strongest imaginable = 96; the adjectives are spaced semi-logarithmically; the LMS only shows adjectives, not numbers, to the subjects.

### Statistical analyses

Arithmetic means of intensity ratings were calculated across the replicates within subjects and were used for statistical analyses. One way ANOVA analyses were carried out and a comparison of means was done using a Tukey’s multiple comparisons test, at a level of significance set at *P* value < 0.05.
